# Cyclic AMP efflux through MRP4 regulates actin dynamics signalling pathway and sperm motility in bovines

**DOI:** 10.1038/s41598-020-72425-5

**Published:** 2020-09-24

**Authors:** Nicolás Chiarante, Carlos A. I. Alonso, Jessica Plaza, Raquel Lottero-Leconte, Camila Arroyo-Salvo, Agustín Yaneff, Claudia E. Osycka-Salut, Carlos Davio, Marcelo Miragaya, Silvina Perez-Martinez

**Affiliations:** 1 Universidad Buenos Aires (UBA), Facultad de Medicina, (CONICET-UBA) Centro de Estudios Farmacológicos y Botánicos (CEFYBO), C1121ABG Buenos Aires, Argentina; 2 Department of Pharmacology and Therapeutics, McGill University, Montreal, QC H3G 1Y6 Canada; 3 Facultad de Ciencias Veterinarias, Instituto de Investigación y Tecnología en Reproducción Animal (INITRA), UBA, Buenos Aires, Argentina; 4 Instituto de Investigaciones Farmacológicas (ININFA) (UBA-CONICET), Facultad de Farmacia y Bioquímica, Universidad de Buenos Aires, C1113AAD Buenos Aires, Argentina; 5 Instituto de Investigaciones Biotecnológicas, Universidad Nacional de San Martín (IIIB-UNSAM/CONICET), Campus Miguelete, Avenida 25 de Mayo y Francia, San Martín, B1650HMP Buenos Aires, Argentina

**Keywords:** Biochemistry, Cell biology

## Abstract

Previously we demonstrated that multidrug resistance-associated protein 4 transporter (MRP4) mediates cAMP efflux in bovine spermatozoa and that extracellular cAMP (ecAMP) triggers events associated to capacitation. Here, we deepen the study of the role of MRP4 in bovine sperm function by using MK571, an MRP4 inhibitor. The incubation of spermatozoa with MK571 during 45 min inhibited capacitation-associated events. MRP4 was localized in post-acrosomal region and mid-piece at 15 min capacitation, while at 45 min it was mainly located in the acrosome. After 15 min, MK571 decreased total sperm motility (TM), progressive motility (PM) and several kinematic parameters. The addition of ecAMP rescued MK571 effect and ecAMP alone increased the percentage of motile sperm and kinematics parameters. Since actin cytoskeleton plays essential roles in the regulation of sperm motility, we investigated if MRP4 activity might affect actin polymerization. After 15 min capacitation, an increase in F-actin was observed, which was inhibited by MK571. This effect was reverted by the addition of ecAMP. Furthermore, ecAMP alone increased F-actin levels while no F-actin was detected with ecAMP in the presence of PKA inhibitors. Our results support the importance of cAMP efflux through MRP4 in sperm capacitation and suggest its involvement in the regulation of actin polymerization and motility.

## Introduction

Mammalian spermatozoa must undergo a series of physiological changes, collectively known as capacitation, in the female reproductive tract in order to make them competent to reach and fertilize the oocyte^1,2^. The biochemical changes associated with sperm capacitation include an efflux of cholesterol from the plasma membrane, changes in protein phosphorylation and protein kinase activity, increases in bicarbonate concentration, Ca^2+^ and cyclic adenosine monophosphate (cAMP) levels, hyperpolarization, among others^3,4^. All these biological processes lead to the induction of an asymmetrical ‘hyperactivated’ motility, and to the ability to undergo the acrosome reaction^3,5^. Those changes are possible thanks to the interplay between cytoskeletal protein complexes and plasma membrane elements, such as ion channels and transporters^6^. Dysfunction of some of these proteins or their regulation mechanisms can cause defects in motility, potentially leading to infertility^7,8^.

Sperm motility is one of the most important characteristics associated with sperm fertilizing ability, as it is required for spermatozoa to travel through the viscous fluids of the female reproductive tract and reach the oocyte. The movement of the spermatozoa’s tail consists of a symmetrical beating that allows the cell to travel with a progressive movement. Effective flagellar movement is dependent on axonemal function and proper ion homeostasis within the flagellar compartment, which is in contact with bicarbonate produced by the male's seminal glands and the female's reproductive fluids^9,10^.

Multidrug resistance-associated protein 4 (MRP4, also known as ABCC4) has been described in somatic cells as an endogenous transporter of several physiological substrates including cyclic nucleotides, with a remarkable affinity for cAMP^11^. In previous works, we demonstrated the presence of MRP4 in the tail of mouse spermatozoa^12^ and in the post acrosomal region and mid-piece of bull spermatozoa^13^. Interestingly, knockout mice for MRP4 are subfertile and present altered testosterone levels and slightly smaller litter than wild type mice^14^. However, characterization of the gametes from these mice was not carried on.

We have also investigated the role of cAMP efflux through MRP4 in sperm capacitation in these two species. In accordance, we have described that extracellular cAMP is an inductor of bovine sperm capacitation^15^ although its role is not clear in the mouse gamete^12^. cAMP is a cyclic nucleotide involved in numerous signal transduction pathways in different reproductive processes such as spermatogenesis, sperm maturation and sperm capacitation^4^. The importance of cAMP in the regulation of sperm motility has been described in mammals^16,17^ and particularly in bovines, a species in which cAMP not only increases the frequency of flagellar beat but also the percentage of motile cells^18,19^.

The phosphorylation / dephosphorylation regulation of proteins is known to be essential for sperm capacitation and motility. In particular, the cAMP-dependent protein kinase or protein kinase A (PKA) pathway is one of the most important metabolic pathways involved in the regulation of sperm motility^20–22^. PKA activity regulation, as well as its subcellular localization, are crucial for compartmentalization of its effect. After activation, PKA binds A-kinase anchor proteins (AKAP) and phosphorylates motility regulatory proteins in the principal piece of the tail. The resulting net phosphorylation in spatial and temporal compartmentalization in flagella depicts the sperm motility status adequately. It is known that serine / threonine kinase activity correlates with increased motility in human and bull^23,24^ whereas other evidences indicate that low-levels of PKA activity regulate progressive motility in mammals^25^. In addition, under physiological conditions, PKA has been identified as the main regulator of actin polymerization^26^. The relevance of this pathway in reproduction has been proven as genetically modified mice not expressing the catalytic (C_s_) subunit of PKA were found to be infertile^27^.

Actin represents one of the main components of the cytoskeleton^28^. The actin state of polymerization and its adequate localization are essential for spermatozoa to acquire fertilizing competence. Polymerization of actin (F-actin) is a requirement for capacitation to take place while rapid actin depolymerization (G-actin) is necessary for the acrosomal reaction^29^. In addition, actin polymerization in the sperm tail during capacitation is essential to modulate motility^30^.

Previous reports indicate that MRP4 activity regulates not only cAMP levels but also PKA activity and the actin polymerization process in human trabecular meshwork cells^31^ and in fibroblasts^32^. Considering the importance of cAMP/PKA/actin polymerization pathways on sperm function, in the present study we aimed to elucidate the role of cAMP efflux through MRP4 and extracellular cAMP in the regulation of sperm motility in bovines. Moreover, the involvement of extracellular cAMP in regulating actin polymerization and PKA activation was studied. In this work, cryopreserved sperm was employed given its economic relevance for livestock production since it is usually used for improving the performance of different assisted reproductive technologies, such as, in vitro fertilization, intracytoplasmic sperm injection or artificial insemination.

## Results

### Role of MRP4 on sperm capacitation

**Figure 1:**
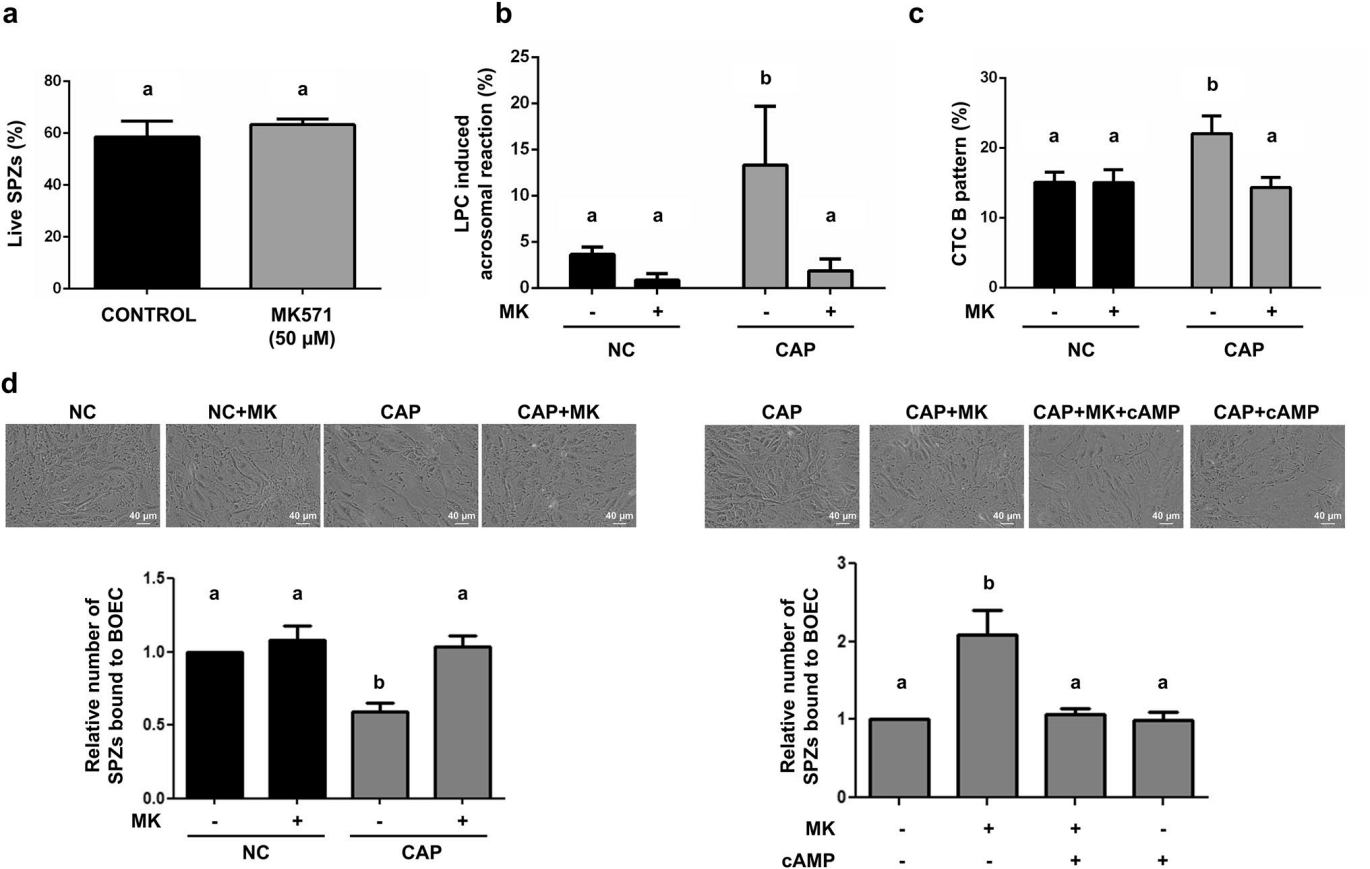
Effect of MK571, an MRP4 inhibitor, on sperm capacitation. Bovine spermatozoa (SPZs) were incubated in non-capacitating (NC) or capacitating (CAP) conditions in the presence or absence of MK571 (50 μM). After 45 min, samples were incubated or not for 15 min with 5 μM l-α-lysophosphatidylcholine (LPC) to induce acrosomal reaction. Cell viability was assessed with Hoechst 33258 (2 μg/ml, 5 min incubation). Cells were fixed, permeabilized and stained with PSA-FITC in order to evaluate acrosomal reaction. At least 200 spermatozoa per condition were examined with a fluorescence microscope (magnification 1,000 ×). (**a**) Live spermatozoa were identified as those without a bright and homogeneous signal in its head. n = 3, p > 0.05. Each n corresponds to a different straw from a different bull. (**b**) Percentage of capacitation was estimated as the difference between the number of live and reacted sperm in the presence of LPC and the number of alive cells spontaneously reacted. n = 3, p < 0.05. Each n corresponds to a different straw from a different bull. (**c**) In a similar way, after 45 min spermatozoa were incubated with 500 μM chlortetracycline (CTC) and examined with a fluorescence microscope. The percentage of cells with a capacitated pattern (B pattern) was quantified. n = 3, p < 0.05. Each n corresponds to a different straw from a different bull (**d**) Spermatozoa were co-incubated with a monolayer of bovine oviductal epithelial cells (BOECs). After 60 min, cells were washed and incubated or not with MK571 (50 μM). Spermatozoa release from the monolayer was induced with NC or CAP medium, supplemented or not with MK571 (50 μM) and cAMP (10 nM). After fixation, photographs were taken (upper panel, magnification 200 ×) and the number of bound sperm was quantified in at least 20 fields of 0.11 mm^2^ (lower pannel). n = 6, p < 0.001 (left panel) and p < 0.005 (right panel). Each n corresponds to a different straw from a different bull. Different letters indicate statistically significant differences.

Previous results from our laboratory employing probenecid, a broad spectrum MRP inhibitor, suggested the involvement of the high-affinity cAMP efflux pump MRP4 in sperm capacitation in bovines^13^. In the present work, we evaluated some capacitation-associated events in the presence of MK571, a more selective inhibitor towards MRP4. The absence of a cytotoxic effect due to sperm incubation with MK571 (50 μM) is shown in Fig. 1a. Results revealed that the induction of sperm capacitation by 40 mM bicarbonate 0.3% BSA in sp-TALP medium (CAP) was prevented by the presence of MK571 (Fig. 1b,c, respectively). In another experimental approach, we analyzed sperm capacitation by the evaluation of sperm release from oviductal cells in bovine oviduct epithelial cells (BOEC)-sperm co-cultures incubated with different treatments. As expected, a decrease of bound sperm to the oviductal cells was observed in the CAP condition with respect to non-capacitating medium (NC). The presence of MK571 prevented sperm release from BOEC (Fig. 1d). Interestingly, when co-cultures were incubated with 10 nM cAMP in addition to MK571, the proportion of bound sperm decreased significantly with respect to cells incubated only with MRP4 inhibitor (Fig. 1d).

### Localization of MRP4 through capacitation

**Figure 2:**
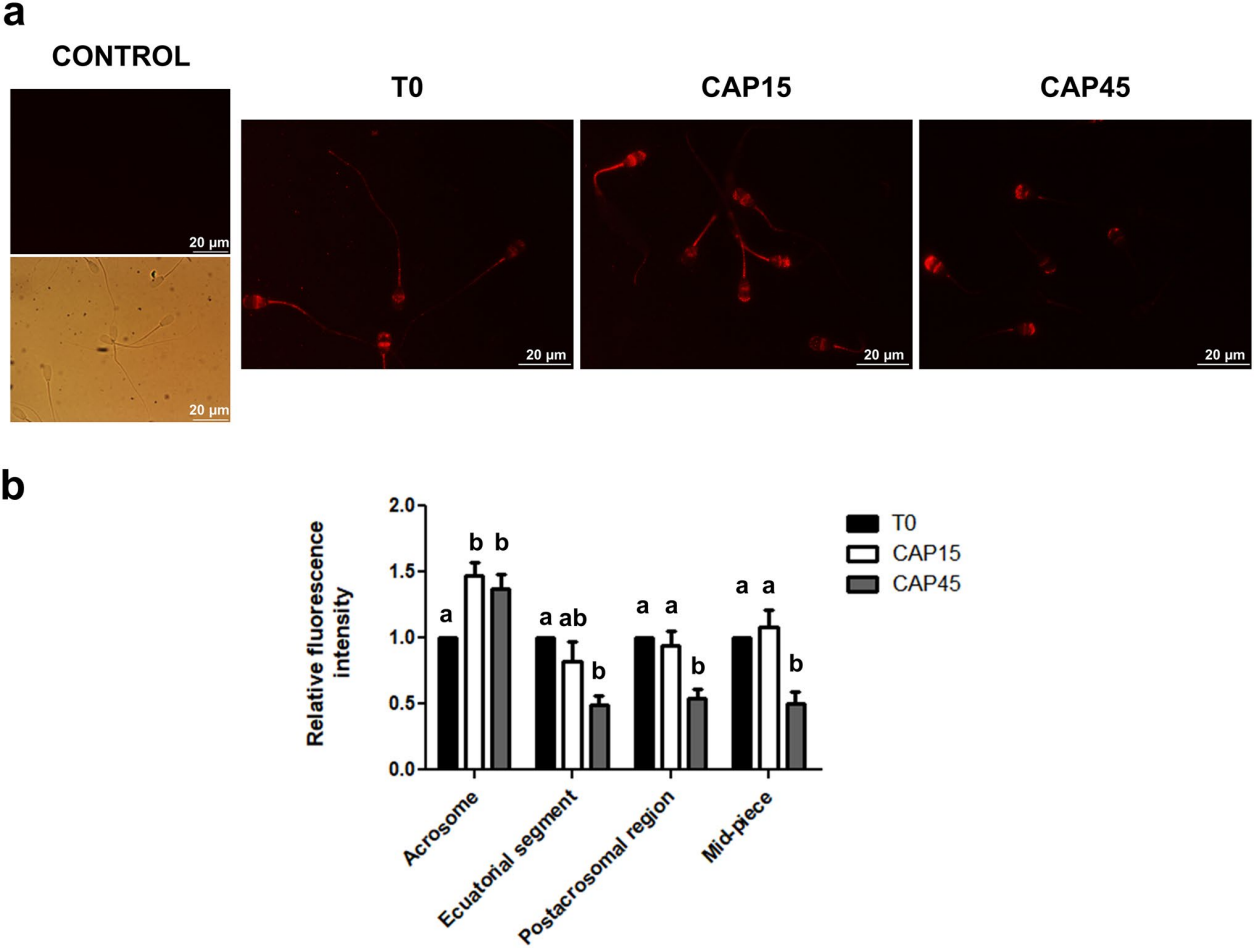
Evaluation of MRP4 localization through capacitation. Cryopreserved bovine sperm were thawed and selected by wool glass columns (T0) and capacitated for 15 or 45 min (CAP15 and CAP45, respectively). An indirect immunofluorescence assay was performed in order to detect MRP4. (**a**) Slides were examined with a fluorescence microscope (magnification 1,000 ×). Control condition represents cells incubated with secondary antibody without primary antibody. Fluorescence and bright field images are displayed for this condition. (**b**) MRP4 presence was quantified as fluorescence intensity relative to the area of each cellular compartment. Fluorescence intensity in each region of the spermatozoa was normalized to the T0 condition. At least 200 spermatozoa were examined for each condition. n = 4, p < 0.05 (acrosome), p < 0.05 (equatorial segment), p < 0.05 (postacrosomal region), p < 0.005 (mid-piece). Each n corresponds to a different straw from a different bull. Different letters indicate statistically significant differences.

Next, we evaluated MRP4 localization at different in vitro capacitation times. Immunofluorescence assays revealed that immediately after thawing (T0), MRP4 mainly localized in midpiece and in the post-acrosomal region including the equatorial segment (Fig. 2a), as had been reported previously by Osycka-Salut et al. 2014. After 15 min of capacitation, in addition to the localization in the post-acrosomal region and midpiece, a significant increase of MRP4 staining (fluorescence intensity) was detected in the acrosomal region (Fig. 2b). At 45 min MRP4 was predominantly observed on the acrosomal region with a decreased signal in the post-acrosomal and mid-piece regions (Fig. 2b).

### Effect of MRP4 inhibition and non-permeable cAMP on sperm motility

**Figure 3:**
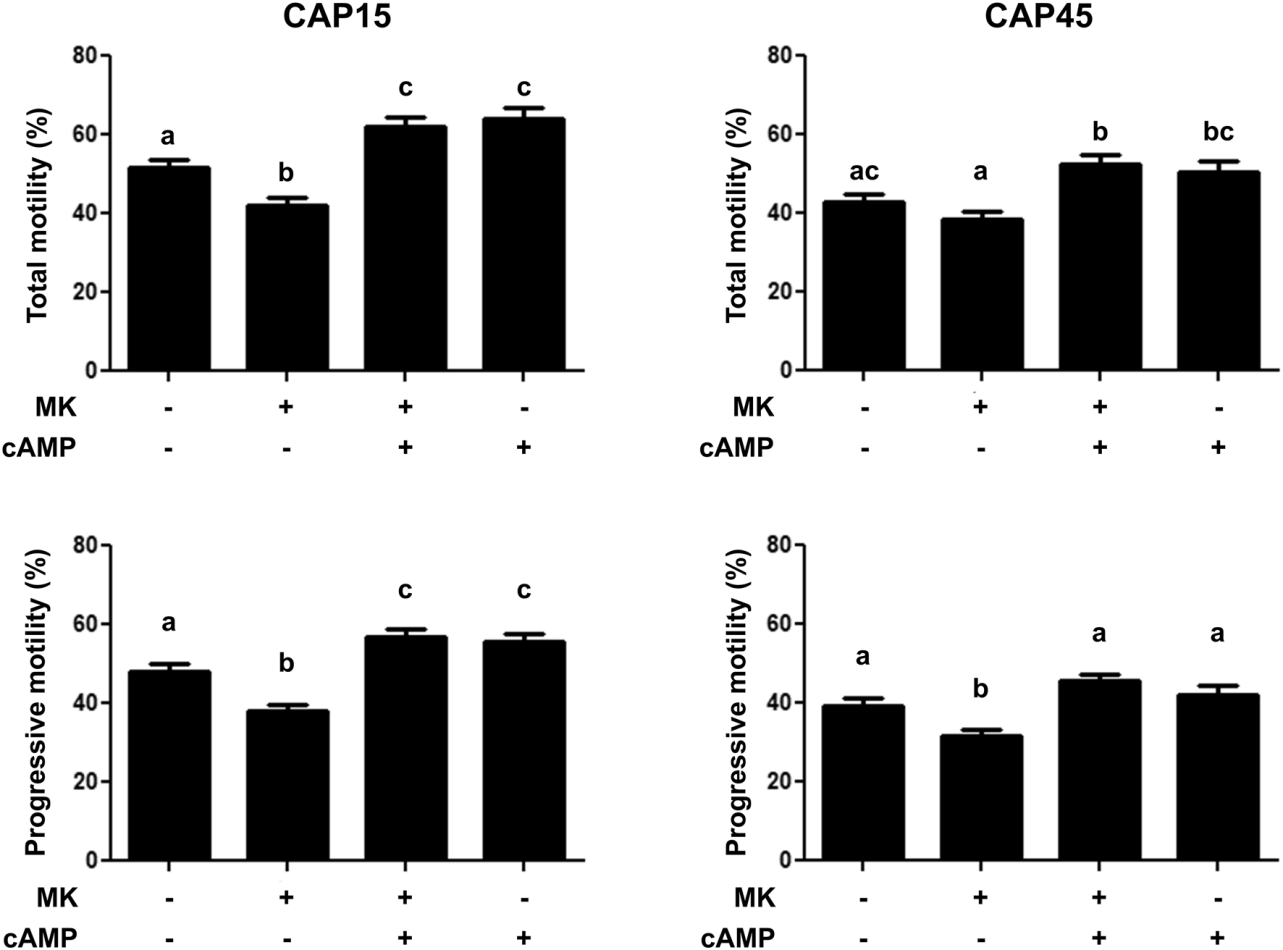
Effect of MK571 and non-permeable cAMP on sperm motility. Cryopreserved bovine sperm were thawed and incubated for 15 or 45 min in CAP conditions with or without MK571 (50 μM) or non-permeable cAMP (10 nM). Spermatozoa were examined with a Computer-assisted Sperm Analysis (CASA) system set for bovine sperm. Total and progressive motility were assessed in 5 videos for each condition. n = 7, p < 0.05 in all cases. Each n corresponds to a different straw from a different bull. Different letters indicate statistically significant differences.

Since we confirmed the presence of MRP4 in the sperm tail at T0 and 15 min of capacitation, we investigated the involvement of this protein on sperm motility. As shown in Fig. 3, after 15 min incubation, MK571 significantly decreased the percentage of total motile (TM) and progressively motile (PM) spermatozoa. In contrast, at 45 min, MK571 affected PM but not TM. Then, we observed that non-permeable cAMP rescued the effect of MK571 on TM and PM at both incubation times (Fig. 3). Moreover, the incubation with cAMP alone significantly increased the percentage of TM and PM cells with respect to CAP condition only at 15 min incubation (Fig. 3). Furthermore, a decrease of kinematic parameters such as VCL, VAP, STR, ALH and BCF was observed in spermatozoa incubated with MK571 at 15 min of capacitation with respect to those cells incubated in CAP conditions (Table 1). At 45 min only BCF was altered with respect to the CAP condition (Table 2). When extracellular cAMP was added to spermatozoa incubated with MK571 for 15 min, BCF reverted to CAP values, while VCL, VSL, VAP, STR and ALH parameters increased with respect to this condition. At 45 min, significant differences were observed in VCL, VAP, ALH and BCF between CAP + MK571 and CAP + MK571 + cAMP conditions. Regarding the evaluation of the effect of cAMP alone, even though the addition of this nucleotide increased many kinematic parameters quantified with respect to CAP condition after 15 min capacitation (VCL, VAP, LIN, STR and ALH) (Table 1), only a significant increase of ALH was observed after 45 min incubation (Table 2).

**Table 1:** Effect of MK571 and non-permeable cAMP on kinematic parameters 15 min after capacitation induction.

15 min	CAP	CAP + MK	CAP + MK + cAMP	CAP + cAMP
VCL	97.9 ± 3.5^a^	78.0 ± 3.5^b^	124.1 ± 3.7^c^	114.8 ± 5.3^c^
VSL	39.8 ± 1.9^ab^	33.3 ± 1.4^a^	51.1 ± 2.4^c^	47.6 ± 3.0^bc^
VAP	48.6 ± 1.9^a^	40.9 ± 1.9^b^	63.0 ± 2.1^c^	59.0 ± 3.0^c^
LIN	40.2 ± 0.9^a^	42.1 ± 1.3^ab^	41.7 ± 0.9^ab^	45.1 ± 1.4^b^
STR	67.5 ± 1.0^a^	66.1 ± 1.4^a^	72.2 ± 1.1^b^	73.4 ± 1.9^b^
ALH	1.02 ± 0.04^a^	0.85 ± 0.04^b^	1.38 ± 0.05^c^	1.28 ± 0.06^c^
WOB	50.0 ± 0.9^a^	51.3 ± 1.3^a^	51.9 ± 0.08^a^	53.4 ± 2.2^a^
BCF	8.30 ± 0.44^a^	6.84 ± 0.29^b^	8.83 ± 0.34^a^	8.33 ± 0.29^a^

Cryopreserved bovine sperm were thawed and incubated for 15 in CAP conditions with or without MK571 (50 μM) or non-permeable cAMP (10 nM). Spermatozoa were examined with a Computer-assisted Sperm Analysis (CASA) system set for bovine sperm. The following kinematic parameters were evaluated: curvilinear velocity (VCL), straight line velocity (VSL), mean path velocity (VAP), linearity (LIN), straightness (STR), amplitude of lateral head displacement (ALH), Wobble coefficient (WOB) and beat cross frequency (BCF). n = 7, p < 0.05 in all cases. Each n corresponds to a different straw from a different bull. Different letters indicate statistically significant differences.

**Table 2:** Effect of MK571 and non-permeable cAMP on kinematic parameters 45 min after capacitation induction.

45 min	CAP	CAP + MK	CAP + MK + cAMP	CAP + cAMP
VCL	80.2 ± 3.2^ab^	71.6 ± 3.6^a^	91.7 ± 4,0^b^	93.4 ± 5.4^b^
VSL	35.4 ± 1.7^a^	34.1 ± 2.2^a^	41.7 ± 3.1^a^	40.0 ± 2.9^a^
VAP	42.0 ± 1.9^ab^	39.8 ± 2.3^a^	50.3 ± 3.0^b^	50.7 ± 3.3^b^
LIN	43.5 ± 0.9^a^	44.2 ± 1.1^a^	45.8 ± 1.4^a^	46.8 ± 1.5^a^
STR	69.3 ± 1.2^a^	68.7 ± 1.4^a^	72.2 ± 1.3^a^	71.3 ± 1.3^a^
ALH	0.88 ± 0.05^ab^	0.77 ± 0.05^a^	1.07 ± 0.04^bc^	1.10 ± 0.07^c^
WOB	52.3 ± 0.9^a^	53.2 ± 1.2^a^	54.8 ± 1.3^a^	54.9 ± 1.0^a^
BCF	7.45 ± 0.31^a^	6.28 ± 0.25^b^	7.63 ± 0.36^a^	7.82 ± 0.48^a^

Cryopreserved bovine sperm were thawed and incubated for 45 in CAP conditions with or without MK571 (50 μM) or non-permeable cAMP (10 nM). Spermatozoa were examined with a Computer-assisted Sperm Analysis (CASA) system set for bovine sperm. The following kinematic parameters were evaluated: curvilinear velocity (VCL), straight line velocity (VSL), mean path velocity (VAP), linearity (LIN), straightness (STR), amplitude of lateral head displacement (ALH), Wobble coefficient (WOB) and beat cross frequency (BCF). n = 7, p < 0.05 in all cases. Each n corresponds to a different straw from a different bull. Different letters indicate statistically significant differences.

### F-actin modulation during capacitation of cryopreserved bovine spermatozoa

Actin polymerization is involved in regulating sperm motility^33,34^. Therefore, we investigated if MRP4 inhibition by MK571 might be affecting actin dynamics and hence sperm motility. In order to confirm if the Alexa Fluor 488-phalloidin probe could be effectively employed to visualize F-actin in cryopreserved bovine sperm, we used Latrunculin B, an actin polymerization inhibitor. Indeed, when spermatozoa were incubated with Latrunculin B (10 μM) in CAP conditions, a significant decrease in F-actin levels was perceived (Supplementary Fig. S1a, upper panel). After analyzing at least 200 spermatozoa, this decrease was confirmed quantitatively (Supplementary Fig. S1a, lower panel). In addition, the effect of actin polymerization inhibition on sperm motility was assessed. As shown in Supplementary Fig. S1b, incubation with Latrunculin B increased the population of immotile spermatozoa and decreased TM and PM.

**Figure 4:**
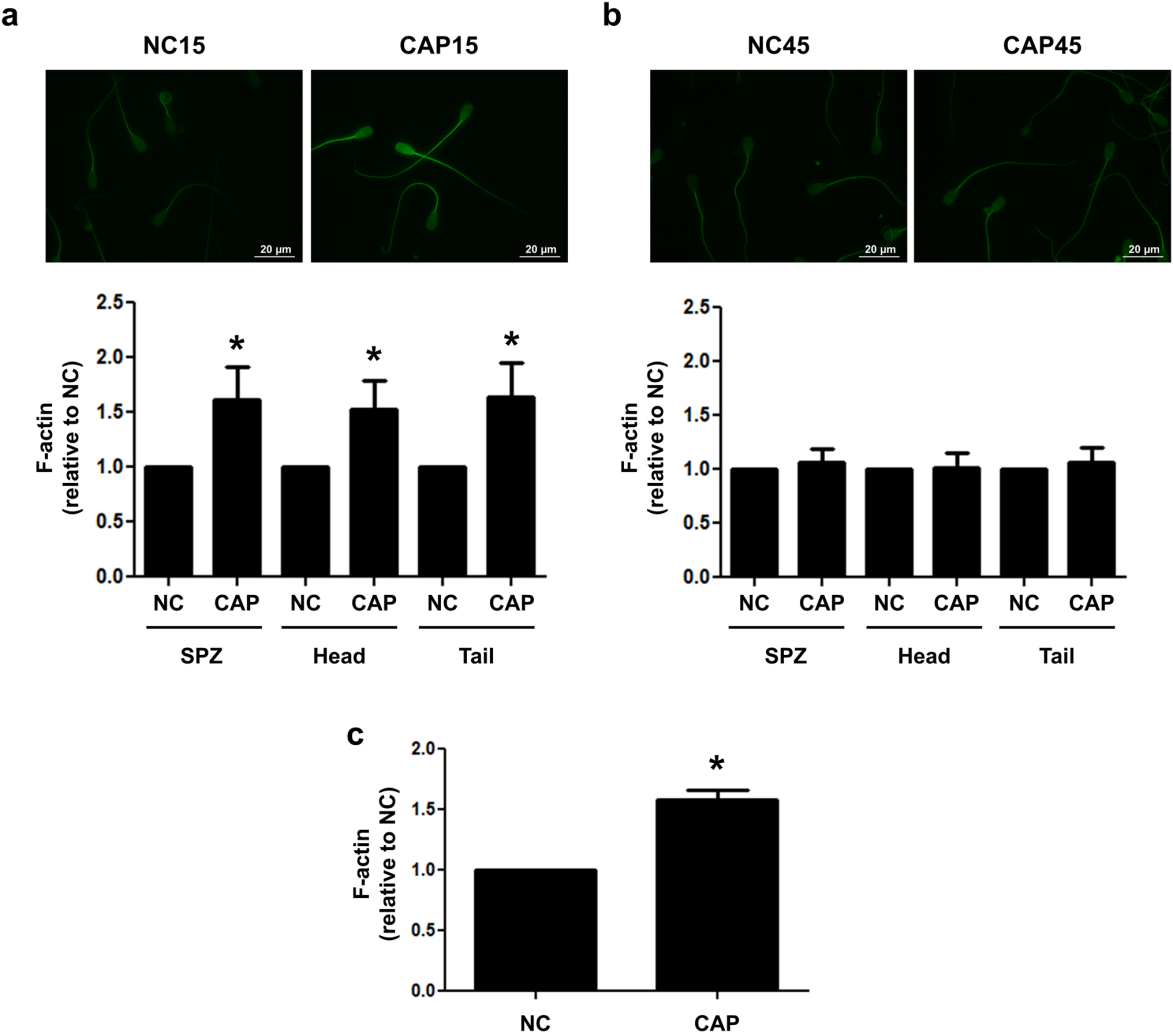
F-actin modulation during capacitation of cryopreserved bovine spermatozoa. Cryopreserved bovine sperm were thawed and incubated in NC and CAP conditions. After 15 (**a**) or 45 min (**b**) incubations, cells were subjected to Alexa Fluor 488-phalloidin staining (6.6 μM, 1 h) and examined with a fluorescence microscope (upper panels, magnification 1,000 ×). The fluorescent signal of F-actin was quantified with the Fiji software for the entire cell, its head or its tail (lower panels). F-actin levels were quantified as fluorescence intensity relative to the area of each cellular compartment and normalized to the NC condition. At least 200 spermatozoa were examined for each condition. n = 5, *p < 0.05 significantly different from NC spermatozoa. Each n corresponds to a different straw from a different bull. (**c**) Alternatively, after 15 min, cells were fixed, permeabilized and incubated with Alexa Fluor 488-phalloidin (6.6 μM, 60 min) and the examined with a flow cytometer. FL1 medians were obtained and normalized to the NC condition. n = 3, *p < 0.05 significantly different from NC spermatozoa. Each n corresponds to a different straw from a different bull.

After that, the kinetics of actin polymerization in cryopreserved bovine sperm was analyzed at 15 and 45 min of incubation. While sperm cells incubated for 45 min did not show differences in F-actin levels between NC and CAP conditions (Fig. 4b), an increase in the amount of F-actin was perceived in sperm incubated for 15 min in CAP conditions with respect to those incubated in NC conditions (Fig. 4a). A significant difference in the fluorescence intensity was quantified on whole cells as well as in their heads and tails, separately. The increase of F-actin levels after 15 min capacitation was also confirmed by flow cytometry (Fig. 4c). Since most effects of MK571 on sperm motility and actin polymerization were observed at 15 min capacitation, the next experimental designs were performed only at this incubation time.

### Effect of MRP4 inhibition and non-permeable cAMP on actin polymerization

**Figure 5:**
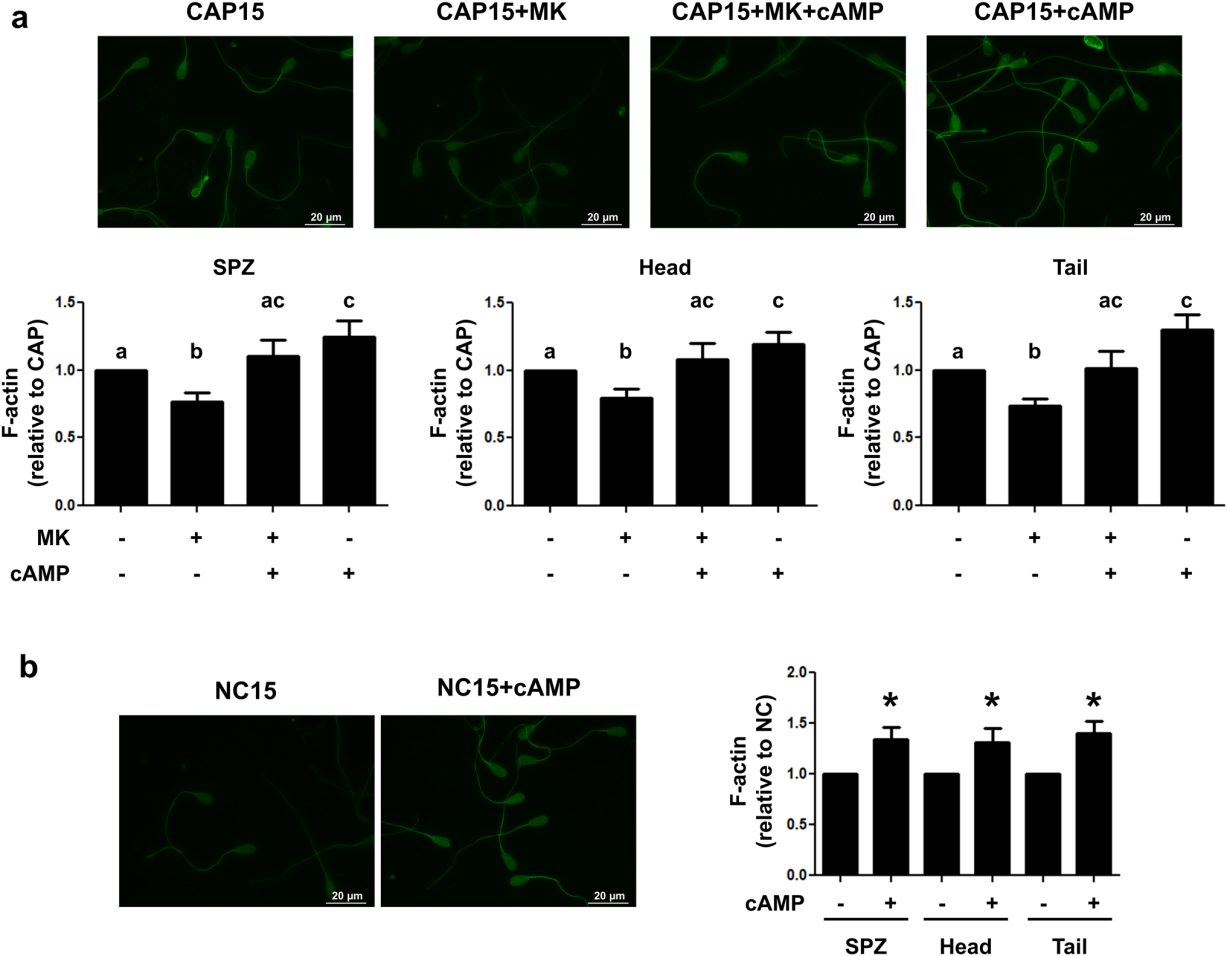
Effect of MK571 and extracellular cAMP on actin polymerization. (**a**) Cryopreserved bovine sperm were thawed and incubated for 15 min in CAP conditions in the presence or absence of MK571 (50 μM) and cAMP (10 nM). (**b**) Alternatively, spermatozoa were incubated in NC medium for 15 min with or without cAMP (10 nM). Cells were then fixed, permeabilized and incubated with Alexa Fluor 488-phalloidin (6.6 μM, 60 min). Cells were examined with a fluorescence microscope (upper panel, magnification 1,000 ×) and the fluorescent signal was quantified with the Fiji software for the entire cell, its head or its tail (lower panel). F-actin levels were quantified as fluorescence intensity relative to the area of each cellular compartment and normalized to the CAP (**a**) or NC (**b**) condition. At least 200 spermatozoa were examined for each condition. n = 6, *p < 0.05 significantly different from sperm incubated in the absence of cAMP. Each n corresponds to a different straw from a different bull. Different letters indicate statistically significant differences.

Next, we evaluated if MRP4 inhibition could alter actin dynamics in bovine spermatozoa. As shown in Fig. 5a, when spermatozoa were capacitated for 15 min in the presence of MK571, actin polymerization was impaired. A quantitative analysis revealed that MRP4 inhibition lead to decreased amounts of F-actin in the whole cell as well as in its head and tail. Based on these findings, we next studied if exogenous cAMP administration could revert the effect of MRP4 inhibition. When the non-permeable nucleotide was administered, a significant increase in F-actin levels was observed (Fig. 5a).

**Figure 6:**
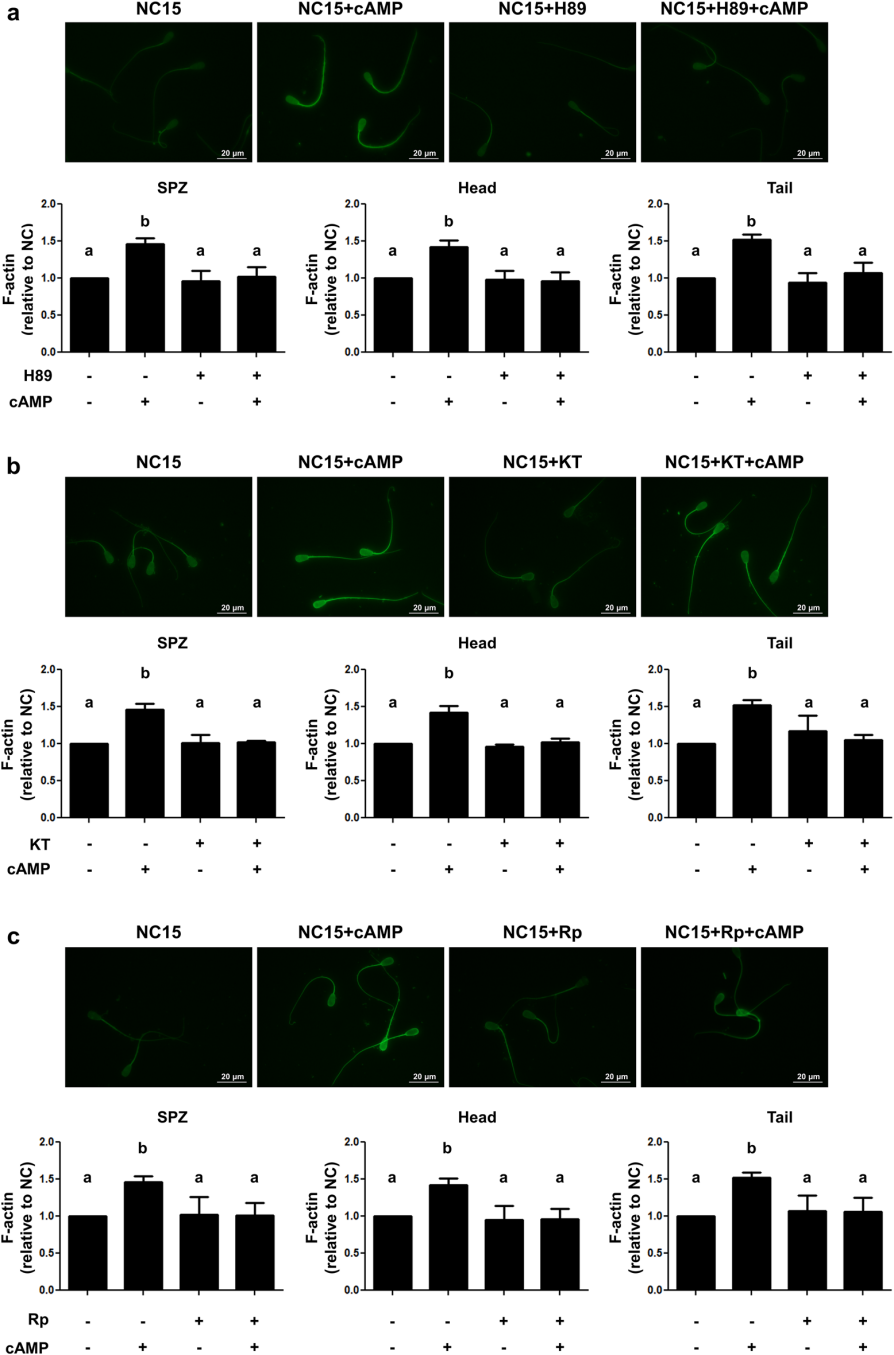
Role of PKA in actin polymerization induced by non-permeable cAMP. Cryopreserved bovine sperm were thawed and incubated for 15 min in NC conditions in the presence or absence of 50 µM H89 (**a**); 100 nM KT5720 (**b**) or 1 mM Rp cAMPS (**c**). The F-actin staining technique was performed with Alexa Fluor 488-phalloidin (6.6 μM, 60 min) and cells were examined with a fluorescence microscope (upper panels, magnification 1,000 ×). The fluorescent signal was quantified with the Fiji software for the entire cell, its head or its tail (lower panel). F-actin levels were quantified as fluorescence intensity relative to the area of each cellular compartment and normalized to the NC condition. At least 200 spermatozoa were examined for each condition. n = 3, *p < 0.05 in all cases. Each n corresponds to a different straw from a different bull. Different letters indicate statistically significant differences.

Taking into account that the incubation with cAMP alone had increased the percentage of motile sperm with respect to CAP condition (Fig. 3), we examined if the addition of non-permeable cAMP might also affect F-actin levels. Indeed, increased actin polymerization was detected in spermatozoa incubated in the presence of the nucleotide (Fig. 5a). Interestingly, we observed that cAMP alone also produced an increase of F-actin in spermatozoa incubated for 15 min in NC conditions (Fig. 5b). When spermatozoa were incubated with the PKA inhibitor H89 (50 µM), the addition of cAMP did not increase F-actin (Fig. 6a). Similar results were obtained when PKA inhibitors, KT5720 (100 nM) and Rp cAMPs (1 mM) were employed (Fig. 6b,c, respectively).

## Discussion

Cyclic AMP is a key regulator in reproductive processes^4^, although its role has been predominantly studied intracellularly. Several reports describe its involvement in critical capacitation outcomes such as hyperactivation and the ability to undergo the acrosome reaction^35–37^. Therefore, the enzymes that regulate cAMP availability play a critical role in male gamete physiology. It has been reported that seminal plasma and oviduct fluids contain nucleotides and phosphodiesterases, which could modulate cAMP homeostasis in the female reproductive tract^38–40^. In sperm, cAMP levels are tightly regulated both temporally and spatially by different types of adenylyl cyclases and phosphodiesterases^41–43^. In fact, lack of soluble adenylyl cyclase (sAC) produces infertility in mice, as sperm are immotile^44^. In turn, one of the best characterized cAMP molecular targets is PKA, and previous reports indicated that the PKA catalytic subunit is only present in the sperm flagellum^45,46^. A link between PKA activity and intracellular cAMP regulation through MRP4 has been suggested in fibroblasts, since it has been demonstrated that MRP4 blockade promotes focal PKA stimulation by punctual cAMP accumulation^32^. Given that it has been postulated that cAMP could act as an important extracellular stimulus in certain cell types^47^, our group has focused on the study of cAMP homeostasis in spermatozoa, examining the role of the cAMP efflux in sperm function. The importance of cAMP efflux through MRP4 has been previously established in bull sperm capacitation^13,15^. This activity of the transporter not only regulates cAMP intracellular levels but also provides the nucleotide to the extracellular space. Interestingly, we also found that extracellular cAMP reverted the inhibitory effect on capacitation produced by probenecid, a broad spectrum MRP inhibitor^13^. In the present work, we used MK571 inhibitor to evaluate the involvement of cAMP efflux by MRP4 in key events implicated in the acquisition of sperm competence in bovines. Consistently with our previous work, MK571 inhibited different events associated to bovine sperm capacitation. We demonstrated that MRP4 impairment inhibited LPC-induced acrosomal reaction as well as a physiological event like sperm release from BOECs. In accordance with previous results^15^, the addition of non-permeable cAMP overcame the effect of the MRP4 inhibitor.

The interaction between sperm and the environment generated by the female reproductive tract is essential to trigger sperm motility. In this sense, several signaling pathways have been described as having a role in mammalian sperm motility. In this work, we found that MRP4 inhibition also affected sperm motility and several kinematic parameters. Our results indicated that sperm population incubated in the presence of MK571 decreased the percentage of TM and PM. This was more evident at 15 min than 45 min incubation. We found that after a 15 min capacitation some kinematic parameters, such as VCL, VAP, ALH and BCF, were decreased when MRP4 was inhibited. VCL, VAP, and also VSL, are strongly associated with flagellar kinetics or propulsive sperm movement^48^, while ALH and BCF express the amplitude and speed of head movement^49^. It should be noted that PM, VCL and VAP are considered among some of the most successful predicting parameters of bull semen fertility^50^. We also observed that extracellular non-permeable cAMP reverted MK571-induced inhibition on those kinematic parameters, as well as on TM and PM.

During capacitation, the motility pattern of the sperm changes from progressive to hyperactivated^51^. Structural organization of ion channels and pumps is critical for the modulation of the dynamic processes associated with the acquisition of the asymmetrical hyperactivated motility^52^. This asymmetrical hyperactivated motility typically displays lower linearity and increased lateral head displacement and curvilinear velocity. Previous work from our group showed that non-permeable cAMP induces bovine sperm hyperactivation after a 45 min capacitation^15^. Although hyperactivation was not directly assessed in this work, the evaluation of the three main kinematic parameters associated with hyperactivation (VCL, ALH and LIN) suggests that a similar result was obtained when cAMP was employed as a stimulus, as VCL and ALH increased greatly and LIN was slightly modified. Accordingly, the opposite effect was perceived when cells were incubated with MK571. As a result, we confirm that cAMP homeostasis regulates bull sperm motility and in addition in this work we show the relevance of cAMP levels at the beginning of capacitation (15 min).

Despite the importance of actin organization and dynamics, little is known about the function and regulation of actin in sperm. The widespread localization of actin along the sperm cell, including equatorial, post-acrosomal region and tail, indicates its crucial role in capacitation, acrosomal exocytosis and motility^53^. Several works support the importance of the proper regulation of actin dynamics for acquisition of sperm fertilizing competence. Indeed, low levels of F-actin in human spermatozoa inhibits motility^30^. Moreover, inhibition of actin polymerization leads to a decrease in the motility of human and murine sperm^33^.

Nevertheless, the actin polymerization dynamics in bovine sperm has only been addressed in fresh sperm, although in vitro fertilization and artificial insemination are mainly performed employing cryopreserved sperm. In ejaculated bull sperm, actin polymerization during capacitation has been assessed up to 4 h, obtaining increasing amounts of F-actin with time^54^. In the present work, we observed an increase in F-actin levels in capacitated cryopreserved bull sperm after 15 min capacitation, and a decrease in polymerized actin 30 min later. Similar actin polymerization dynamics displaying a peak has been described in other species such as guinea pig^55^.

The actin cytoskeleton might be involved in the migration of mitochondria to the midpiece during spermiogenesis and in providing a scaffold that confines mitochondria to this compartment^56^. In support of this hypothesis, genetically modified male mice lacking the actin-interacting protein nectin-2 are infertile and, although sperm motility appeared to be normal, long-term periods of incubation in capacitating medium led to a loss of motility in vitro and reduced migration of the sperm into the oviduct in vivo^57^. These results highlight the importance of the actin cytoskeleton in the midpiece for normal sperm function and fertility. In this sense, our results suggested that the presence of MRP4 in midpiece might associate with F-actin structures/actin cytoskeleton and regulate flagellar movement. Therefore, we investigated the role of MRP4 in the regulation of actin dynamics establishing that the inhibition of MRP4 activity decreases sperm populations showing F-actin. This suggests a key role of cAMP efflux through MRP4 in the regulation of the F-actin structure. Further studies will be required to elucidate if the pathway connecting MRP4 and extracellular cAMP with actin polymerization involves well-known actin regulators such as Rho GTPases, gelsolin, cofilin and many others. Noteworthy, a similar regulation of actin polymerization through MRP4 has been described in Human Trabecular Meshwork cells^31^. As previously mentioned, MRP4 has also been identified to control cell movement in fibroblasts through the regulation of cAMP levels, PKA activity and actin polymerization^32,58^.

It should be mentioned that the role and importance of MRP4 and purinergic signalling might be species specific, as differences between mouse and bovine spermatozoa have been observed. This is evidenced by the fact that inhibition of capacitation associated events by MRP4 blockade is overcome when cAMP is added to the incubation media in bovine but not in murine spermatozoa^12^. This suggests that the purpose of the nucleotide extrusion in bovine spermatozoa is primarily to provide cAMP to the extracellular space, whereas in murine spermatozoa cAMP efflux by MRP4 might solely be a key regulator of intracellular cAMP levels/PKA activity. This is also supported by the difference in MRP4 localization between these species, which may result in association with different molecules triggering, therefore, different signalling pathways^12,13^. In this sense, the relocalization of MRP4 to the acrosomal region during bovine sperm capacitation suggests a role for this transporter in events related to acrosomal reaction, a topic worth further exploration.

Previous results from Alonso et al. suggest the ability of extracellular cAMP to trigger the broad and complex signaling events necessary for the acquisition of sperm competence in bovines. Results from this work strongly support the fact that the nucleotide exerts responses from the extracellular space, as we showed that the increase of motile sperm by non-permeable cAMP was mediated by PKA activity and actin polymerization. This is in accordance with previous findings from our laboratory that showed a rise of sAC/PKA pathway activity induced by extracellular cAMP^15^. Furthermore, the increase of PKA-phosphorylation levels by extracellular cAMP was detected in the midpiece of bovine sperm^15^. Accordingly, it is known that PKA targets are mainly located in the sperm tail^25^ and that sAC/cAMP/PKA pathway is important for motility acquisition^22^.

In conclusion, results presented in this work indicate that cAMP efflux through MRP4 regulates sperm motility in bull spermatozoa. Furthermore, the signaling pathway triggered by MRP4 activity and extracellular cAMP is required to maintain actin dynamics for flagellar movement. These data support previous results from our laboratory, suggesting that exclusion of the nucleotide to the extracellular space might achieve spatial and temporal cAMP compartmentalization, necessary for capacitation-associated events to take place. Moreover, the hypothesis of the role of extruded cAMP in an autocrine/paracrine fashion in bovine sperm is reinforced in this work.

## Materials and methods

### Chemicals

MK571, H89, cAMP, RP-Adenosine 3′,5′-cyclic monophosphorothioate triethylammonium salt (Rp cAMPS), chlortetracycline (CTC), Hoechst 33258, *Pissum sativum* agglutinin-FITC (PSA-FITC), l-α-lysophosphatidylcholine (LPC) and bovine serum albumin (BSA) were acquired from Sigma-Aldrich (MO, USA). KT5720 was purchased from Tocris Bioscience (Bristol, UK). Latrunculin B (Lat B) was acquired from Cayman Chemical (MI, USA). Monoclonal antibody anti-MRP4 and anti-rabbit IgG coupled to Alexa Fluor 555 were obtained from Cell Signaling Technology (MA, USA) and Abcam (Cambridge, UK) respectively. Alexa Fluor 488-phalloidin, M199 medium, gentamicin and fungizone were purchased from Invitrogen (CA, USA). All other chemicals were of analytical grade and obtained from standard sources.

### Sperm preparation

Straws of frozen bovine semen (20–25 × 10^6^ spermatozoa/ml) were kindly provided by Centro de Reproducción Bovina San Antonio de Areco (CRB), Centro de Inseminación Artificial La Elisa (CIALE) and Cooperativa de Inseminación Artificial Venado Tuerto (CIAVT). Straws were thawed for 30 s in a water bath at 38.5 °C. Sperm were selected by the wool glass column method as previously described^59^, and washed by centrifugation in BSA-free Tyrode’s Albumin Lactate Pyruvate (sp-TALP). Finally, pellets were resuspended in BSA-free sp-TALP and assessed for motility and sperm concentration using a hemocytometer mounted on a microscope stage heated at 38.5 °C.

### In vitro sperm capacitation

Ten × 10^6^ spermatozoa/ml were incubated in non-capacitating (NC) medium (sp-TALP: 99 mM NaCl; 3.1 mM KCl; 0.4 mM NaH_2_PO_4_; 0.4 mM MgCl_2_.6H_2_O; 21.6 mM sodium lactate; 10 mM HEPES; 2 mM CaCl_2_.H_2_O; 25 mM NaHCO_3_; 1 mM sodium piruvate; 50 mg/ml gentamycin; pH 7.37)^60^ or capacitating (CAP) medium (0.3% BSA and 40 mM NaHCO_3_ sp-TALP) at 38.5 °C and 5% CO_2_ atmosphere for 15 or 45 min^61^. This CAP medium has previously shown to be adequate to achieve capacitation and cAMP extrusion^13^. In some experiments, cells were co-incubated with cAMP (10 nM), an MRP4 inhibitor (50 µM MK571), PKA inhibitors (50 µM H89; 100 nM KT5720 or 1 mM Rp cAMPS) or an F-actin assembly inhibitor (10 µM Latrunculin B). The same cAMP and MK571 concentrations were employed as in previous works from our group^13,15^.

### Viability assay

Spermatozoa were incubated in the presence or absence of MK571 (50 μM) for 45 min. Then, samples were incubated with Hoechst 33,258 (2 μg/ml, 5 min). Spermatozoa were fixed and examined with a Nikon Eclipse E200 (Tokyo, Japan) fluorescence microscope (magnification 1,000 ×) coupled to a DS-Fi1 Nikon photographic camera. Live sperm were identified as those without a bright and homogeneous signal in its head. At least 200 spermatozoa per condition were evaluated.

### Assessment of sperm capacitation

Capacitation was assessed by different techniques: l-α-lysophosphatidylcholine (LPC)-induced acrosome reaction/*Pissum sativum* agglutinin (PSA)-FITC staining, chlortetracycline (CTC) assay and evaluation of sperm release from bovine oviductal epithelial cells (BOEC).

The induction of the acrosome reaction was performed as previously described^13^. Spermatozoa were incubated in NC or CAP conditions in the presence or absence of MK571 (50 μM). After 45 min, samples were incubated or not for 15 min with LPC (5 μM) to induce acrosomal reaction. Cell viability was assessed with Hoechst 33,258 (2 μg/ml, 5 min incubation). Spermatozoa were fixed, permeabilized and stained with PSA-FITC in order to evaluate acrosomal reaction. At least 200 cells per condition were examined with a Nikon Eclipse E200 (Tokyo, Japan) fluorescence microscope (magnification 1,000 ×). Capacitation was estimated as the difference between the number of live and reacted spermatozoa in the presence of LPC and the number of live sperm spontaneously reacted. The CTC assay was performed as previously detailed^59^. In a similar way, after 45 min spermatozoa were incubated with CTC (500 μM) and examined with a fluorescence microscope. The percentage of cells with a capacitated pattern (also known as B pattern) was quantified^62^.

### Bovine oviductal epithelia cell cultures and sperm co-cultures

As sperm plasma membranes are remodel during capacitation, spermatozoa detach from the oviductal epithelium. Therefore, we assessed sperm capacitation by the evaluation of sperm release from oviductal cells in BOEC-sperm co-cultures treated with different conditions.

Bovine oviducts were kindly donated from Compañía de Carniceros Sociedad Anónima (COCARSA) slaughterhouse (Buenos Aires, Argentina). Oviductal epithelia cell cultures were prepared as described previously^63^. Briefly, oviducts were collected, transported at 4 °C, cleaned of surrounding tissues and washed three times in sterile PBS at 4 °C. Then, oviducts were isolated, flushed with sterile PBS and squeezed by pressure with tweezers.

Laminae of bovine oviduct epithelial cells (BOECs) from ampulla and isthmus were recovered from different animals and pools of epithelial cells were obtained. After centrifugation at 1,500*g* for 5 min of the different pools of BOECs, cells were incubated in M199 medium supplemented with FBS (10%), gentamicin (0.1 mg/ml) and fungizone (1 μg/ml) at 38.5 °C in a 5% CO_2_ atmosphere. Incubations were performed in six-well tissue culture dishes containing 12 mm round cover slips. After 48 h, BOECs were washed by centrifugation (1,500*g*, 5 min) and placed once again in the tissue dishes. The medium was removed every 48 h. When confluence was achieved, around 7 days after starting the culture, the oviductal monolayers were washed three times in BSA-free sp-TALP and incubated in this medium for 60 min before adding the spermatozoa.

Confluent BOEC monolayers were inseminated with sperm suspensions (0.5 × 10^6^ sperm/ml of BSA-free sp-TALP/well) for 60 min at 38.5 °C in a 5% CO_2_ atmosphere. Then, unbound sperm were removed by washing, and co-cultures were incubated for 15 min in BSA-free sp-TALP supplemented or not with MK571 (50 µM). After washing once again, spermatozoa release from BOEC was stimulated with a 15 min incubation with BSA-free or BSA containing sp-TALP (40 mM NaHCO_3_), supplemented or not with MK571 (50 µM) or cAMP (10 nM) were appropriate. Released spermatozoa were removed and co-cultures fixed in glutaraldehyde (2.5% v/v) for 60 min at room temperature. Cover slips containing the co-cultures were mounted on a glass slide and the number of bound sperm was evaluated by analyzing 20 fields of 0.11 mm^2^/cover slip under a phase contrast microscope (Olympus IMT-2, Tokyo, Japan).

### Immunofluorescence

An indirect immunofluorescence method was performed in order to detect MRP4 as previously described^13^. Spermatozoa were fixed (30 min, room temperature, 0.2% w/v paraformaldehyde) immediately after thawing (T0), and 15 or 45 min after capacitation induction. Cells were immobilized on slides and permeabilized with TPBS-Triton X100 (0.5%) for 20 min at room temperature. Non-specific binding sites were blocked with T-PBS BSA 3% for 1 h and spermatozoa were incubated with 10 mg/ml primary MRP4 antibody. Cells were then incubated for 60 min with anti-rabbit IgG coupled to Alexa Fluor 555 (1:500). Specificity of the immunodetection was assessed by omitting the first antibody. Finally, sperm cells were mounted and evaluated with a Nikon Eclipse E200 (Tokyo, Japan) fluorescence microscope (magnification 1,000 ×). MRP4 presence was quantified in all photographed sperm as fluorescence intensity relative to the area of each cellular compartment. The Fiji software was employed for this task.

### Sperm motility analysis

After 15 or 45 min of sperm capacitation in the presence or absence of MK571 (50 µM) or cAMP (10 nM), sperm motility was evaluated using Computer-assisted Sperm Analysis (AndroVision, Minitüb GmbH, Tiefenbach, Germany) with a negative phase contrast microscope coupled to a heated stage (38 °C). 10 μl aliquots of sperm were pipetted on to a warm glass slide and covered with 18 × 18 mm coverslips. Sperm motility was assessed with the bull semen set-up provided by the manufacturer. For each sample, at least five fields were evaluated. The settings employed were the following: 30 frames acquired, 60 images per second, minimum contrast 70, minimum cell size 8 pixels, intensity 0.62–1.32, non-motile head size 12 pixels, non-motile intensity 80, threshold straightness 80, frame rate of 60 Hz, magnification 200 ×, and cell size of 30–170 μm^2^. The following parameters were measured: total motility (TM), progressive motility (PM), curvilinear velocity (VCL), straight line velocity (VSL), mean path velocity (VAP), linearity (LIN), straightness (STR), amplitude of lateral head displacement (ALH), Wobble coefficient (WOB) and beat cross frequency (BCF).

Alternatively, experiment shown in Supplementary Fig. S1 was performed counting spermatozoa manually. Cells were evaluated in an Olympus IMT-2 microscope coupled to a 60D BODY photographic camera (Canon, Tokyo, Japan). Recorded videos were analyzed and spermatozoa were classified as non-motile (NM), not progressively motile (NPM) and progressively motile (PM). Total motile (TM) spermatozoa were quantified as NMP + PM. At least 200 cells per condition were evaluated.

### Assessment of F-actin

In order to assess F-actin levels, the Alexa Fluor 488-phalloidin probe was employed in two different experimental approaches: fluorescence microscopy and flow cytometry.

Spermatozoa were incubated in NC or CAP conditions for 15 or 45 min, in the presence or absence of MK571 (50 µM) or cAMP (10 nM). In some experiments, cells were co-incubated with Latrunculin B (10 µM), H89 (50 µM), KT5720 (100 nM) or Rp cAMPs (1 mM). Then, cells were fixed for 60 min at room temperature employing a PBS solution containing 0.1% glutaraldehyde and 1.5% formaldehyde. Cells were then washed, incubated 15 min in a 25 mM NH_4_Cl solution and placed on slides. A 7 min incubation with acetone at − 20 °C was performed for permeabilization to occur, followed by extensive washing. Spermatozoa were afterwards incubated for 60 min with 6.6 µM Alexa Fluor 488-phalloidin to stain F-actin. Phalloidin is a bicyclic peptide belonging to a family of toxins isolated from the *Amanita phalloides* mushroom. Its high affinity for F-actin makes it the gold standard method for actin polymerization evaluation^64^. Finally, cells were mounted and examined in a Nikon Eclipse E200 (Tokyo, Japan) fluorescence microscope (magnification 1,000 ×). The Fiji software was employed for image analysis and quantification.

Alternatively, F-actin was assessed in a BD Accuri cell cytometer (BD Biosciences, CA, USA) equipped with a 533/30 nm filter for FITC/Alexa Fluor 488 detection. Side-scatter area (SSC-A) and forward-scatter area (FSC-A) data were collected from 15,000 events per sample in order to define sperm populations. Singlets were selected from a forward-scatter height (FSC-H) vs. FSC-A dot plot and replotted in a FL1 histogram. Results show the mean of Alexa Fluor 488 median fluorescence.

### Statistical analysis

All values are expressed as mean ± S.E.M. of at least three different experiments. Raw data were analyzed by Shapiro–Wilks and F tests to assess normality of data distribution and variance homogeneity, respectively. Statistical analysis of the data was performed by using Student's t-test or one-way analysis of variance (ANOVA) followed by Bonferroni post-hoc tests when appropriate. p < 0.05 denotes a statistically significant difference. The GraphPad Prism 5.0 software was employed in all cases.

## Supplementary information

Supplementary Figure S1.
