# Supplementary material for: Cyclic AMP efflux through MRP4 regulates actin dynamics signalling pathway and sperm motility in bovines
Source: Sci Rep. 2020 Sep 24;10:15619. doi: 10.1038/s41598-020-72425-5 (PMC7518284; doi:10.1038/s41598-020-72425-5)
Supplement: Supplementary file 1 — Supplementary Figure S1. [file 41598_2020_72425_MOESM1_ESM.pdf]

CYCLIC AMP EFFLUX THROUGH MRP4 REGULATES SPERM MOTILITY IN BOVINES: ROLE OF EXTRACELLULAR CYCLIC AMP IN ACTIN DYNAMICS SIGNALLING PATHWAY.

Nicolás Chiarante[a], Carlos A. I. Alonso[b], Jessica Plaza[c], Raquel Lottero[a], Camila Arroyo-Salvo[a], Agustín Yaneff[d], Claudia E. Osycka-Salut[e], Carlos Davio[d], Marcelo Miragaya[c], Silvina Perez Martinez[a]*

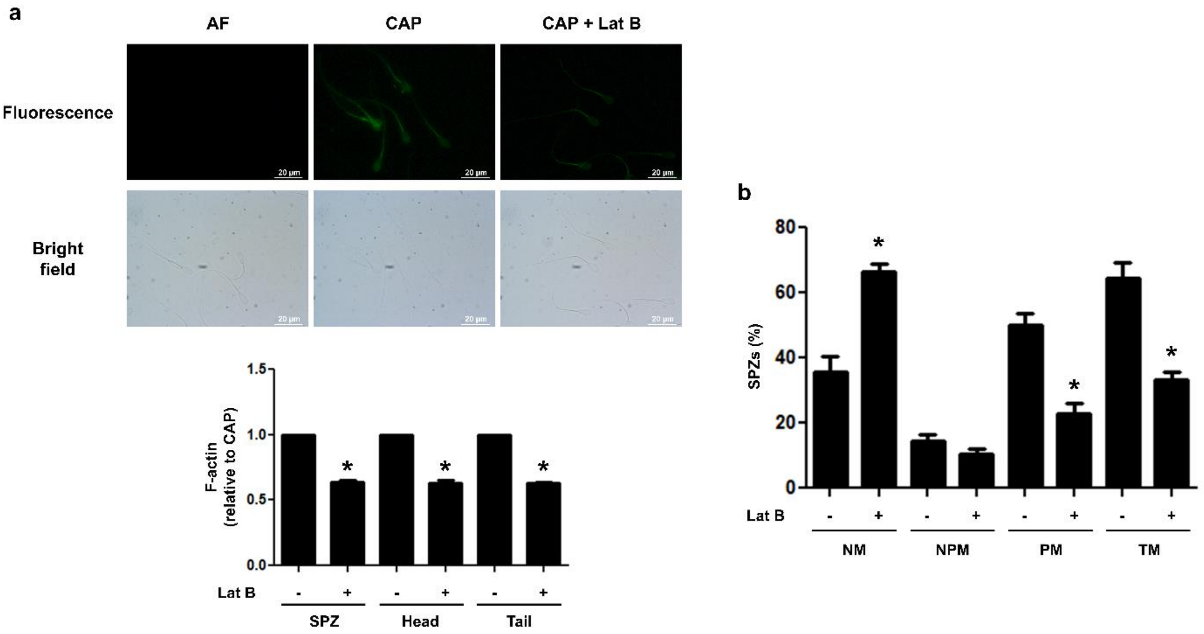

Supplementary Figure S1. Effect of Latrunculin B on actin polymerization and sperm motility. (a) Cryopreserved bovine sperm were thawed and incubated in CAP conditions with or without 10 μM Latrunculin B (actin polymerization inhibitor) for 45 min. After fixation, cells were permeabilized, incubated with Alexa Fluor 488-phalloidin (6.6 μM, 60 min) and finally examined with a fluorescence microscope (upper panel, magnification 1000x). Fluorescent signal was quantified with the Fiji software for the entire cell, its head or its tail (lower panel). F-actin levels were quantified as fluorescence intensity relative to the area of each cellular compartment. Fluorescence intensity in each region of the spermatozoa was normalized to the condition without Latrunculin B. At least 200

spermatozoa were examined in each case. n=3, *$p<0.005$ significantly different from cells incubated in the absence of Latrunculin B. (b) Alternatively, after 45 min incubation, 5 second videos were acquired examining the cells with a phase contrast microscope. Subjective motility was measured from at least 3 videos for each condition. Spermatozoa were classified as non-motile (NM), not progressively motile (NMP) and progressively motile (PM). Total motile (TM) spermatozoa were quantified as NMP+PM. n=3, *$p<0.05$ significantly different from cells incubated in the absence of Latrunculin B.
